# Foraging intention affects whether willow tits call to attract members of mixed-species flocks

**DOI:** 10.1098/rsos.170222

**Published:** 2017-06-07

**Authors:** Toshitaka N. Suzuki, Nobuyuki Kutsukake

**Affiliations:** 1 Center for Ecological Research, Kyoto University, 2-509-3 Hirano, Otsu, Shiga 520-2113, Japan; 2 Department of Evolutionary Studies of Biosystems, SOKENDAI (The Graduate University for Advanced Studies), Hayama, Kanagawa 240-0193, Japan

**Keywords:** flock dynamics, foraging decisions, mixed-species flocks, willow tits, vocal signals

## Abstract

Understanding how individual behaviour influences the spatial and temporal distribution of other species is necessary to resolve the complex structure of species assemblages. Mixed-species bird flocks provide an ideal opportunity to investigate this issue, because members of the flocks are involved in a variety of behavioural interactions between species. Willow tits (*Poecile montanus*) often produce loud calls when visiting a new foraging patch to recruit other members of mixed-species flocks. The costs and benefits of flocking would differ with individual foraging behaviours (i.e. immediate consumption or caching); thus, willow tits may adjust the production of loud calls according to their foraging intention. In this study, we investigated the link between foraging decisions and calling behaviour in willow tits and tested its influence on the temporal cohesion with members of mixed-species flocks. Observations at experimental foraging patches showed that willow tits produced more calls when they consumed food items compared with when they cached them. Playback experiments revealed that these calls attracted flock members and helped to maintain their presence at foraging patches. Thus, willow tits adjusted calling behaviour according to their foraging intention, thereby coordinating the associations with members of mixed-species flocks. Our findings demonstrate the influence of individual decision-making on temporal cohesion with other species and highlight the importance of interspecific communication in mixed-species flocking dynamics.

## 1. Introduction

One of the greatest challenges in ecology and evolution is to understand how individual behaviour influences the spatial and temporal structuring of species assemblages [1–3]. Mixed-species bird flocks provide an excellent system to investigate this issue, because members of the flocks are involved in a variety of behavioural interactions between species, which may influence the flocking dynamics [3–6]. For example, individuals might follow heterospecific individuals to exploit their food items [7–11] or approach them to cooperatively drive off a predator [12–15]. Such organization of multi-species associations would reflect the individual decisions of flock members to maximize their own fitness benefits. However, knowledge is limited about how the decision of individuals is made in multi-species environments and how it influences social cohesion with other species.

**Figure 1.**
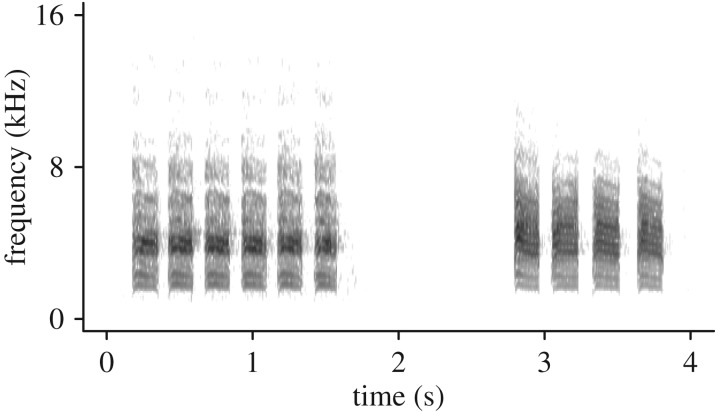
Sound spectrogram of willow tit ‘tää’ calls, consisting of multiple ‘tää’ notes.

Interspecific vocal communication might provide a way of linking the decisions of individuals with the temporal dynamics of mixed-species flocks [3,16]. For example, when separated from mixed-species flocks, greater racket-tailed drongos (*Dicrurus paradiseus*) attract flock members by mimicking the song and contact calls of other species [17]. By actively facilitating the social cohesion with other species, drongos can increase their foraging efficiency by catching insects flying out from the disturbance of other species and also by kleptoparasitism [18,19]. Similarly, in mixed-species flocks in Japan, willow tits (*Poecile montanus*) produce ‘tää’ calls (figure 1) when visiting a new foraging patch alone, which attracts both conspecific and heterospecific members of mixed-species flocks to the food source [20,21]. By recruiting other members of a mixed-species flock, willow tits may reduce the risk of predation [22,23]. However, tits do not typically produce calls when visiting the foraging patch as part of a flock, probably because redundant call production increases the risk of attracting predators [24]. Mixed-species flocks of tits are highly variable in their membership over short periods of time and may not be stable at a single foraging patch [25]. Therefore, the dynamic adjustment of calling behaviour may allow tits to control for the changes in temporal social cohesion with other individuals as well as to manage the immediate risk of predation.

Willow tits may adjust their calling behaviour based on subsequent foraging decisions, as well as the social context, because the costs and benefits of flocking would differ depending on the foraging decision. Willow tits have two different foraging behaviours: (i) eating food items immediately or (ii) caching food items for future use [26]. When handling and consuming food, willow tits might benefit from attracting other flock members, with associations increasing anti-predator benefits (e.g. sharing vigilance) [23]. In contrast, while caching food items, close associations with other flock members might increase the risk of cached food items being pilfered [27–29]. Tits might therefore reduce the risk of pilferage by caching items in highly dispersed locations [30]. As a result, the anti-predator benefits of attracting other individuals to food by calling would be greater for tits when consuming food items than when caching them. Such differences in the advantages of group foraging, in turn, might influence the temporal dynamics of mixed-species flocks.

In this study, we investigated whether the calling behaviour of willow tits was related to their subsequent foraging decisions and if there was a concomitant effect on temporal cohesion with other members within mixed-species flocks. We predicted that willow tits would produce ‘tää’ calls only when they intended to consume a food item and not when they intended to cache the food. By creating artificial foraging patches, we examined how willow tits changed their foraging behaviour on repeated visits to the food source and how they adjusted the production of ‘tää’ calls according to their foraging intention. We also analysed the influence of social context on the production of ‘tää’ calls, since a previous study found that willow tits adjusted their calling behaviour according to the presence or absence of flock members [21]. Although ‘tää’ calls have been shown to function in attracting both conspecific and heterospecific flock members to a foraging patch [21], it remains unclear whether these calls function in maintaining temporal cohesion with other flock members. We conducted playback experiments to test whether these calls serve to attract other flock members and keep them at a foraging patch for longer than that with a silent control. We found that willow tits adjusted their calling behaviour according to their foraging intention and social context, thereby influencing the temporal cohesion with other species involved in mixed-species flocks.

## 2. Material and methods

### 2.1. Study site and subjects

The study was conducted in a mixed deciduous–coniferous forest in Karuizawa, Nagano, Japan (36°19–22′ N, 138°32–37′ E). In this forest, mixed-species flocks contained several passerine and non-passerine species. Out of these species, willow tits, Japanese tits (*Parus minor*), varied tits (*Poecile varius*) and nuthatches (*Sitta europaea*) used artificial feeders with sunflower seeds, and frequently consumed sunflower seeds [21]. There was a noticeable interspecific dominance hierarchy at the foraging sites, with nuthatches being the most dominant species, followed by varied tits, Japanese tits and willow tits [21]. In the present study, willow tits tended to arrive at novel food patches first (50%) (*n* = 54; see below), whereas other species (Japanese tits (33%), varied tits (13%) and nuthatches (4%)) tended to arrive later.

### 2.2. Observations at the artificial foraging patch

To investigate the relationship between the calling and foraging behaviours of willow tits, a forest block (approximately 500 × 500 m) was selected, in which most of the birds (willow tits, Japanese tits, varied tits and nuthatches) were captured using feeder traps and were colour-banded for individual identification during the course of a 3-year field research project. For willow tits, all of the individuals observed in the forest (*n* = 54) were individually identifiable with colour leg bands. Fifty-four forest openings were first chosen within the forest block, which were separated by at least 30 m from each other, and a forest opening was randomly assigned for each trial. Then, a wooden feeder (25 × 25 × 5 cm) was placed on the ground at each site and filled with 300 g sunflower seeds for each trial. The observation location was situated at a distance of 10–12 m from the feeder, which was ideal for collecting data on bird behaviour without causing disturbance [21].

The focal-observation method [31] was used to record the behaviour of individual willow tits. When a willow tit was within 15 m of the feeder, behavioural observations were started. Tits made a calling decision on reaching the tree branches around the feeder. Subsequently, they took a seed from the feeder, and then ate it or cached it (i.e. subsequent foraging behaviour). Tits typically continued to visit the feeder during the first 10 min after their initial arrival; therefore, behavioural observations lasted for approximately 10 min (see also [25]). The following variables were recorded: (i) whether the focal willow tit produced ‘tää’ calls before it took a seed from the feeder, (ii) whether it consumed the seed or cached it, and (iii) whether other flock members were present within a 5 m radius around the focal bird when it visited the feeder. It was often not possible to follow the foraging behaviours of willow tits when they flew a considerable distance away (more than 30 m from the feeder) after taking a seed. These instances (*n* = 26) were considered as caching, since a previous study showed that when flying such a distance, willow tits always cache food items inside tree trunks or branches [26]. Behavioural observations were recorded vocally using an LS370 parabolic microphone (Fuji Planning Co., Tokyo, Japan) connected to an MZ-RH1 Hi-MD Walkman (sampling wave files at 44.1 kHz, 16 bits; Sony Co., Tokyo, Japan).

Up to five trials (separated by at least 1 h) were conducted daily, resulting in 54 trials from 26 January to 9 February 2008. Data from 13 sites, in which willow tits never visited the feeder within the first 10 min of the arrival of the other species, were excluded from the analysis. In these trials, willow tits were not observed from the observation position; thus, they appeared to engage in foraging at a far distance. Therefore, the final dataset included data from 41 trials only. No experimental sites were used more than once to ensure that the birds visited novel foraging patches in all trials. All trials were conducted under calm and dry weather conditions from 08.00 to 15.00 h (Japan Standard Time). A total of 381 feeder visitations (up to 10 visitations in each trial) were recorded for 13 different willow tits that had unique combinations of colour-bands.

### 2.3. Playback experiments

Playback experiments were conducted to test whether the ‘tää’ calls of willow tits function to maintain temporal cohesion with other flock members. Playback stimuli were constructed from our recording libraries of 10 different willow tits. A single call composed of four ‘tää’ notes was chosen from each source individual. This call was repeated at a rate of one call per 6 s to create a 60 s calling bout. Thus, playback stimuli for call treatment were created so as to be typical of natural calling bout of tää’ calls produced in a food context [20,21]. The 60 s calling bout was preceded by a 30 s silent period and then these sounds were repeated 10 times, resulting in a 15 min (900 s) sound file. Background noise was used as a control stimulus, because it allowed us to assess the stability of mixed-species flocks without any playback of the call. Background noise was chosen from the same files for call playbacks, and was edited in the same way as the playback call files, i.e. a 90 s sound (60 s background noise preceded by a 30 s silent period) was repeated 10 times to fill a 15 min sound file. Call files were prepared for playing at a standardized volume (72 dB at 1 m from a loud speaker, measured using an SM-325 sound level meter; AS ONE Corporation, Osaka, Japan). Background noise files were prepared for broadcasting at the same amplitude as the background noise level in call playbacks (50 dB at 1 m).

Trials were conducted from 15 to 19 December 2015. First, we searched for a mixed-species flock in the forest. When a flock was found and a willow tit was seen, a loudspeaker was placed at the base of a tree. It was ensured that the loudspeaker was placed within approximately 15 m of the willow tit. Then, the observer moved to the observation location at 15 m from the loudspeaker, which was estimated using a tape measure, and the playback of either a call or a background noise was started. During the silent periods of each trial, the number of individuals within 15 m distance of the loudspeaker was counted, because, at this distance, all birds were visible from the observation position. This scanning method is generally acceptable for measuring the behavioural response of groups [31]. Trials were conducted at 20 sites, which were separated by at least 400 m, because previous observations of colour-banded individuals showed that this distance is enough to ensure that independent data are collected from different individuals [21]. The order of calls and background noise was alternated and counterbalanced across sites, so that call A at site 1, background noise A at site 2, background noise B at site 3, call B at site 4, and so on (capital letters correspond to the identity of the original recording files). Unique exemplars were used for each trial to avoid pseudoreplication [32].

### 2.4. Statistical analysis

All statistical analyses were carried out in R for Mac v. 3.1.2 [33]. Generalized linear mixed models (*glmer* function in the package *lme4* [34]) with a binomial error structure and logit-link function were used to analyse foraging and calling behaviours. In the first model, the foraging behaviour (consume or cache) was modelled in response to the number of feeder visitations and presence or absence of other flock members. In the second model, calling behaviour (yes or no) was modelled in response to the number of feeder visitations, presence or absence of flock members, and subsequent foraging behaviour (consume or cache). In both models, the presence or absence of any flock members was included regardless of their species identity, because comparisons of Akaike's information criterion (AIC) values supported the view that this categorization better explains the adjustment of foraging and calling behaviour in willow tits; the models with the presence or absence of any flock members had smaller AIC values (first model: 282.8; second model: 241.9) compared with those including the presence or absence of each species of bird separately (first model: 287.9; second model: 243.6). For both models, the fixed terms were tested for possible multicollinearity by examining the variance inflation factor (VIF; function *vif* in the package *DAAG* [35]). The highest VIFs in the first and second models were 1.03 and 1.26, respectively, which were less than the cut-off value of 10 [36]. In addition, the maximum correlation coefficients between fixed terms were generally low for both models (first model: −0.16; second model: 0.24). Therefore, there was no multicollinearity effect on the fixed terms. All fixed terms were standardized using *z*-transformation to facilitate direct comparisons of the effect sizes before the analyses [37]. Since we collected data from 13 individual willow tits in 41 trials, all of the models included the individual identity of focal bird and the individual trials as random terms.

Non-parametric statistics were used to analyse the data of the playback experiment. Mood's median tests were used to investigate whether birds were more likely to approach the loudspeaker with the playback of ‘tää’ calls than that with the playback of background noise, and whether they continued foraging for longer around the loudspeaker when exposed to calls than to background noise. All tests were two-tailed, and the statistical level was set at *α* = 0.05.

## 3. Results

### 3.1. Factors affecting foraging decisions

**Figure 2.**
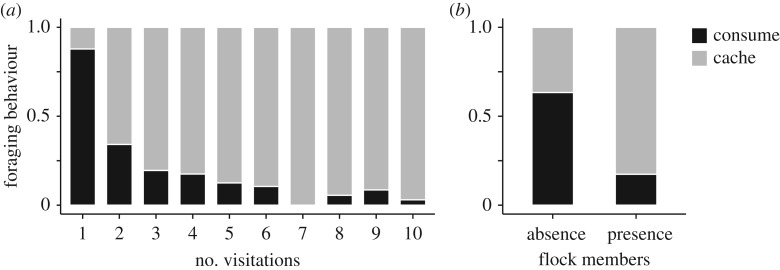
Factors affecting the foraging behaviours (consumption or caching) by willow tits. (*a*) Willow tits shifted their foraging modes from consumption to caching as they repeated feeder visitations. (*b*) Willow tits generally consumed seeds when they visited the feeder alone, but they tended to cache a seed in the presence of flock members. Sample size: *n* = 381 feeder visitations by 13 individuals in 41 trials.

Willow tits changed their foraging behaviours when repeatedly visiting a feeder (table 1). For early visits, they consumed a seed close to the feeder. In later visits, they became more likely to carry seeds and cache them away from the feeder (figure 2*a*). Social context also affected the foraging behaviours of willow tits; they tended to consume seeds when they visited the feeder alone, whereas they typically cached the seeds when other flock members were already present around the feeder (figure 2*b* and table 1).

**Table 1.** Factors affecting the foraging behaviour of willow tits analysed using the generalized linear mixed model. The foraging behaviour (consume or cache) was modelled in response to the number of repeated visits to the feeder and presence or absence of flock members. s.e.: standard error. Sample size: *n* = 381 feeder visitations by 13 individuals in 41 trials. All fixed terms were standardized before the analysis.

model	estimate	s.e.	d.f.	*χ*^2^	*p*
intercept	−1.85	0.35			
number of feeder visitations	−1.6	0.23	1	77.97	<0.001
flock members (presence/absence)	−0.44	0.15	1	9.12	<0.01

### 3.2. Factors affecting calling behaviour

**Figure 3.**
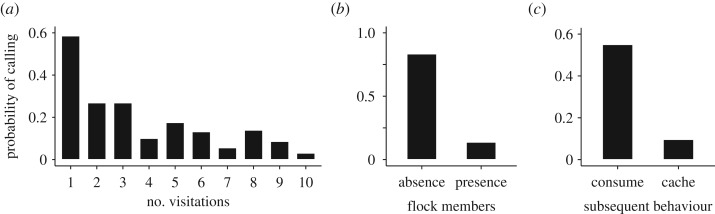
Factors affecting the production of ‘tää’ calls by willow tits. (*a*) The probability of focal tits producing calls declined as they repeated feeder visitations. (*b*) The probability of calling declining due to the presence of flock members. (*c*) The probability of calling being affected by subsequent foraging behaviour. Sample size: *n* = 381 feeder visitations by 13 individuals in 41 trials.

Willow tits changed their calling behaviour during the repeated visitations to the feeder (table 2). They frequently produced ‘tää’ calls on their first access to the feeder, but after the second visit to the feeder, they reduced their probability of calling (figure 3*a*). Social context affected the ‘tää’ calling in willow tits; they typically produced calls when visiting the feeder alone, whereas they rarely vocalized when other flock members were present (figure 3*b* and table 2). Foraging intention also had a significant effect on the calling behaviour of willow tits; they produced ‘tää’ calls particularly before they consumed a seed close to the feeder, whereas they typically did not call before caching a seed (figure 3*c* and table 2).

**Table 2:** Factors affecting the production of ‘tää’ calls by willow tits analysed using the generalized linear mixed model. Calling behaviour (yes or no) was modelled in response to the number of repeated visits to the feeder, subsequent foraging behaviour (consume or cache), and presence or absence of flock members. s.e.: standard error. Sample size: *n* = 381 feeder visitations by 13 individuals in 41 trials. All fixed terms were standardized before the analysis.

model	estimate	s.e.	d.f.	*χ*^2^	*p*
intercept	−2.67	0.44			
number of feeder visitations	0.97	0.21	1	7.52	<0.01
flock members (presence/absence)	−0.64	0.25	1	15.33	<0.001
subsequent foraging behaviour (consume/cache)	−0.86	0.26	1	25.21	<0.001

### 3.3. Playback experiments

**Figure 4.**
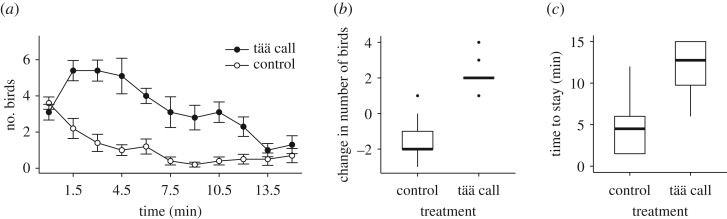
Responses of birds to the playback of ‘tää’ calls of willow tits and background noise. (*a*) Number of birds (mean ± s.e.) within a 15 m distance of the loudspeaker. (*b*) Changes in the number of birds during the first 1.5 min of the playback. (*c*) Time to stay within a 15 m distance of the loudspeaker after the beginning of the playback. Sample size: *n* = 10 for both treatments.

Playback experiments showed that ‘tää’ calls attracted both conspecific and heterospecific flock members to the loudspeaker, and maintained the cohesion of flock members at a foraging patch (figure 4*a*). During the first 1.5 min of the playback, more birds approached during the playback of the ‘tää’ calls than when the background noise was played (figure 4*b*; Mood's median test: *p* < 0.001). Willow tits (6/10 trials), Japanese tits (6/10 trials) and coal tits (4/10 trials) were the most common species that approached the ‘tää’ calls during the first 1.5 min of playback. The time that birds continued foraging within a 15 m distance of the loudspeaker was significantly longer during the playback of ‘tää’ calls than the background noise (figure 4*c*; Mood's median test: *p* = 0.023), indicating that calls facilitated temporal cohesion with flock members at the foraging patch.

## 4. Discussion

Willow tits produced ‘tää’ calls when they focused on consuming a food item (figure 3*c*), and these calls were also shown to facilitate social cohesion with both conspecific and heterospecific flock members at a foraging patch (figure 4). In contrast, willow tits rarely produced calls when caching a food item (figure 3*c*). Therefore, the temporal stability of mixed-species flocks at a patch was maintained particularly when willow tits consumed a seed and produced ‘tää’ calls. Attracting other flock members may provide anti-predator advantages to the callers. For example, close associations with other flock members allow individuals to share vigilance costs while foraging [23,38]. In addition, individuals within mixed-species flocks may recognize heterospecific alarm calls, which enhance predator detection and choosing an appropriate anti-predator behaviour [12,13]. Furthermore, even when approached by a predator, an increase in the number of surrounding individuals reduces an individual's probability of being attacked by the predator [22]. These anti-predator benefits of flocking would therefore be larger for willow tits consuming food items close to the feeder than those caching them, because tits caching food typically fly a considerable distance from the feeder [26].

Willow tits change their foraging and calling behaviours according to the temporal social cohesion with other flock members (figures 2*b* and 3*b*). When alone, willow tits typically produce calls and consume seeds close to the feeder. In contrast, when other flock members are present, willow tits silently take and cache the seeds. Although attracting other flock members may allow individuals to reduce the risk of predation [22,23], the production of loud calls, such as ‘tää’ calls, may also attract predators [24]. Therefore, the adjustment of call production under different social environments may serve to manage immediate predation risk. When caching food items, willow tits travel a considerable distance from the feeder in order to reduce the risk of pilferage by other individuals [26,30]. Therefore, willow tits caching seeds alone are expected to be more isolated from the flocks than those caching seeds with other flock members around. This may explain why willow tits rarely cache food items when other flock members are absent around the feeder (figure 2*b*).

In the course of repeated visits to the feeder, willow tits changed their foraging modes and calling behaviour (figures 2*a* and 3*a*). Initially, birds produced calls and consumed seeds when they arrived at a feeder. In later visits they would reduce their calling and take seeds in order to cache. Because the experimental foraging patches were created at novel sites, advertising the location of the feeder by calling might be important for food discoverers to direct other flock members to the patches [21,39]. This result is also consistent with our previous observations that willow tits produce calls more often when they find food items at a permanent feeder alone compared with when other individuals are present [21]. In addition to the calling, feeding around the feeder might aid tits to advertise the exact location of the feeder to other birds. The change in foraging behaviours may also be explained by individuals' physiological condition. In winter, birds lose heat from their body surface, and require more energy to support high metabolic expenditure [28,40]. Therefore, consuming seeds first might allow tits to cover the energy cost of subsequent flights for caching.

The playback of willow tit ‘tää’ calls attracted both conspecific and heterospecific flock members (figure 4*a*,*b*). Since the discovery of profitable foraging patches is difficult and often stochastic [9–11], approaching ‘tää’ calls may allow flock members to find food sources more effectively [16,23]. This is consistent with previous studies showing that parids are often followed by other species that exploit the same food items [4]. In general, parids have large vocal repertoires [41,42] and produce different call types in different contexts, such as when driving away a predator [43–45] or when in flight [46]. These calls may be recognized by other species and evoke behavioural responses [12–14], but the community-level outcomes of interspecific communication remain poorly understood [3]. Uncovering the link between sophistication in communication and its influence on other species may advance our understanding about mechanisms underlying the temporal stability and movement patterns of mixed-species flocks.

This study demonstrates that the foraging decisions of individuals are linked with their calling behaviour, which influences the temporal cohesion with other species involved in mixed-species flocks. Our findings support the view that individuals within mixed-species flocks have the potential to actively coordinate the associations with other species [17,21] and highlight the importance of interspecific vocal signalling in the flock dynamics. Future detailed studies on interspecific social interactions may not only improve our present understanding of the costs and benefits of mixed-species flocking but also provide insight into the evolution of social foraging strategies in mixed-species environments.

## Supplementary Material

Supporting data
